# A Novel Computational Method for Detecting DNA Methylation Sites with DNA Sequence Information and Physicochemical Properties

**DOI:** 10.3390/ijms19020511

**Published:** 2018-02-08

**Authors:** Gaofeng Pan, Limin Jiang, Jijun Tang, Fei Guo

**Affiliations:** 1 School of Computer Science and Technology, Tianjin University, Tianjin 300350, China; pangaofeng@tju.edu.cn (G.P.); jianglm@tju.edu.cn (L.J.); tangjijun@tju.edu.cn (J.T.); 2 Tianjin University Institute of Computational Biology, Tianjin University, Tianjin 300350, China; 3 Department of Computer Science and Engineering, University of South Carolina, Columbia, SC 29208, USA

**Keywords:** DNA methylation, scBS-seq profiled mouse embryonic stem cells, *k*-gram, multivariate mutual information, discrete wavelet transform, PseAAC, Sparse Bayesian learning, support vector machine, feature selection

## Abstract

DNA methylation is an important biochemical process, and it has a close connection with many types of cancer. Research about DNA methylation can help us to understand the regulation mechanism and epigenetic reprogramming. Therefore, it becomes very important to recognize the methylation sites in the DNA sequence. In the past several decades, many computational methods—especially machine learning methods—have been developed since the high-throughout sequencing technology became widely used in research and industry. In order to accurately identify whether or not a nucleotide residue is methylated under the specific DNA sequence context, we propose a novel method that overcomes the shortcomings of previous methods for predicting methylation sites. We use *k*-gram, multivariate mutual information, discrete wavelet transform, and pseudo amino acid composition to extract features, and train a sparse Bayesian learning model to do DNA methylation prediction. Five criteria—area under the receiver operating characteristic curve (AUC), Matthew’s correlation coefficient (MCC), accuracy (ACC), sensitivity (SN), and specificity—are used to evaluate the prediction results of our method. On the benchmark dataset, we could reach 0.8632 on AUC, 0.8017 on ACC, 0.5558 on MCC, and 0.7268 on SN. Additionally, the best results on two scBS-seq profiled mouse embryonic stem cells datasets were 0.8896 and 0.9511 by AUC, respectively. When compared with other outstanding methods, our method surpassed them on the accuracy of prediction. The improvement of AUC by our method compared to other methods was at least 0.0399. For the convenience of other researchers, our code has been uploaded to a file hosting service, and can be downloaded from: https://figshare.com/s/0697b692d802861282d3.

## 1. Introduction

DNA methylation is a “silencing” epigenetic mark [1], and its function closely connects with life development and disease formation [2,3]. Research about DNA methylation can help us understand the regulation of epigenetic reprogramming [4,5,6], gene expression [7], and genome imprinting [8]. When a methyl group is added to the DNA molecule (often to the fifth carbon of a cytosine ring), this cytosine is a 5-methyl-cytosine and the activity of the DNA sequence around it can be changed. The methyl group may affect the transcription of genes [9,10] and may cause tumorigenesis and cancer progression [11]. So, research about DNA methylation can also help disease sub-classification [12] and biomarker identification [13].

Some previous studies have shown that DNA methylation has special sequence patterns [14], and the methylated sites have a strong connection with their sequential contexts [15]. The sequence constitution of a macromolecule like DNA or protein has a great connection to its polymorphism and phenotype [16]. Based on the properties of methylation and the abundant information contained in the sequential context, it is possible to identify whether a DNA sequence is methylated according to its nucleotides composition [17]. Currently, there are several significant research works that have provided computational methods based on simple nucleotide sequences to predict DNA methylation sites [18]. A common way to do this is by applying a machine learning method, using appropriate functions to extract features from the DNA sequence, and then training a classifier by using the sequence’s feature vector. The trained classifier can predict the methylation state of a new sequence.

Those previous methods use different features to train the classifier. Some use sequence information to extract features, while some apply additional structure properties. Functional expression is also used by some researchers as features. Methylator proposed by Bhasin et al. [19] simply converts sequences to feature vectors by using conventional binary sparse encoding. HDFINDER proposed by Das et al. [20] counts the number of specific DNA segments. Liu’s iDNA-Methyl [21] includes physicochemical properties between nucleotides into its features. Bock et al. [22] refer to attributes such as structure, conservation, binding sites, and so on. More comprehensively, Previti et al. [23] combined sequence repeats, structure property, evolutionary conservation, and physicochemical properties together in their method. Other methods also combine several features together to train the classifier. MethCGI proposed by Fang et al. [24] joins CpG ratios, TpG contents, binding sites, and the distribution of Alu Y. CpGIMethPred proposed by Zheng et al. [25] takes CpG island, sequence composition, structure patterns, the distribution of conserved binding sites, and histone methylation status into consideration. In the method proposed by Zhang et al. [26], genome annotation marks and cis-regulatory elements are used as features. Different from the above methods, DeepCpG [27] uses a deep learning model. Because the deep learning model can generate features from the original sequences, it is not necessary to define features in this method. All of these methods have significantly contributed to the methylation problems, yet they still have some shortcomings and can be improved upon to achieve better results.

In the choosing of classifier, most methods use a support vector machine (SVM) algorithm for its great performance on small datasets with parameter adjustment. Methylator [19], HDFINDER [20], iDNA-Methyl [21], MethCGI [24], and CpGIMethPred [25] use SVM to predict methylation states. However, there are also some methods which use other classifiers rather than SVM. The method proposed by Zhang et al. [26] uses random forests to do prediction. The method proposed by Previti et al. [23] uses decision tree instead of SVM. Different classifiers are suited to different problems. Thus, choosing the right classifier is important for a method.

In order to propose an effective computational method for DNA methylation prediction, we combined four feature extraction methods: *k*-gram, multivariate mutual information (MMI), discrete wavelet transform (DWT), and pseudo amino acid composition (PseAAC). The *k*-gram can get an item’s frequency features in a sequence, and has been used in many computational biology problems [28]. MMI can analyze the correlation information between two nucleotides [29]. DWT can capture both the frequency and location information [30]. PseAAC can reflect the covariance between each composition of the sequence [31]. With those features, we can train a classifier and adjust its parameters to achieve the best performance. Then, the trained sparse Bayesian learning model can predict the methylation state of a sample sequence. We tested our method on a benchmark dataset extracted from MethDB [32] and two scBS-seq datasets sequenced from scBS-seq profiled mouse embryonic stem cells (mESCs) [33]. On the benchmark dataset, our method could reach 0.8632 on area under the receiver operating characteristic curve (AUC), 0.8017 on accuracy (ACC), 0.5558 on Matthew’s correlation coefficient (MCC), and 0.7268 on sensitivity (SN). All of those values are better than the other three methods, and the improvements are significant. When compared with the best of the three methods, the AUC of our method was increased by 0.0268, and SN by 0.1143. On another two scBS-seq datasets, our method’s AUCs were 0.8896 and 0.9511, which are better than the other two methods. Results show that our method could effectively predict DNA methylation.

## 2. Method

**Figure 1:**
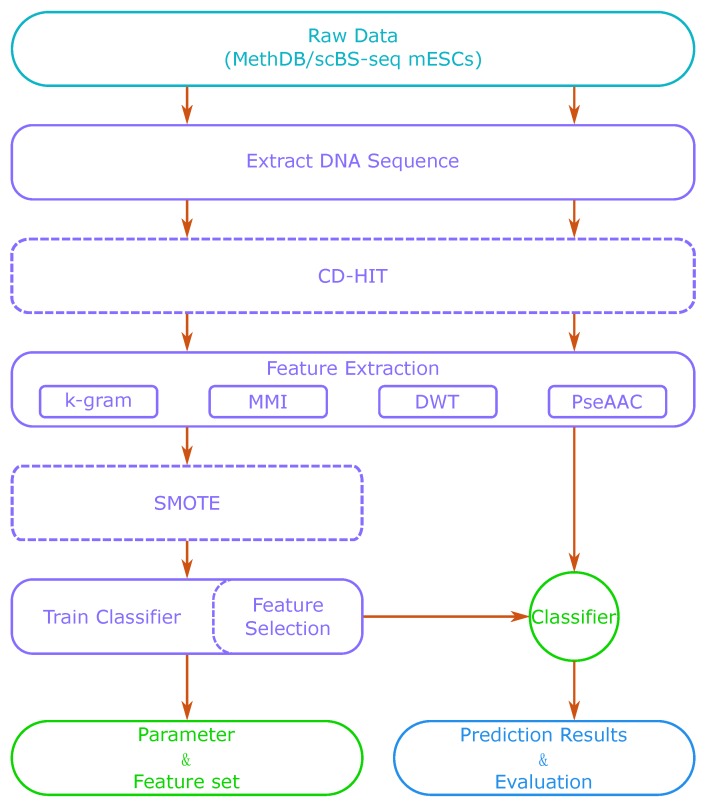
The flow chart of our method. DWT: discrete wavelet transform; mESC: mouse embryonic stem cell; MMI: multivariate mutual information; PseAAC: pseudo amino acid composition; CD-HIT: Cluster Database at High Identity with Tolerance; SMOTE: Synthetic Minority Over-sampling Technique.

We used DNA sequence information and physicochemical properties to distinguish methylated sites and non-methylated sites. The sequence features were extracted by *k*-gram and multivariate mutual information (MMI) [29], considering the nucleotide sequences around the candidate methylation sites. The physicochemical features were extracted by discrete wavelet transform (DWT) [34] and pseudo amino acid composition (PseAAC) [35], which are associated with the physical structure of nucleotide permutations [30,36]. Then, we trained the sparse Bayesian learning model to predict the DNA methylation sites. Figure 1 shows a flow chart of our method. Firstly, we extracted DNA sequences around CpG sites from a sequence database or a reference genome. Then, we used feature extraction methods to generate a feature vector. With those extracted features, we trained a classifier such as SVM or a sparse Bayesian learning model. We also adjusted the model’s parameters to obtain its best performance. After training, we could use the classifier to predict methylation states of new DNA sequences. When the dataset was small, we could also apply CD-HIT (Cluster Database at High Identity with Tolerance) and SMOTE (Synthetic Minority Over-sampling Technique) algorithms in the process to optimize the training set. Feature selection is optional, as it can increase the accuracy of prediction, but is also time-consuming.

### 2.1. Physicochemical Properties

There are 16 2-permutations of nucleotides, namely AA,AC, ⋯,TT. According to a previous research work [37], each permutation has six physicochemical properties (Twist, Tilt, Roll, Shift, Slide, Rise) associated with its physical structure. Table 1 lists the original values of the six physical structural properties adapted from Goni et al. (2007) [37].

Based on the physical structural properties between two nucleotides, we can transform one (l+1)-length nucleotide sequence $L=N_{1}N_{2}\cdots N_{l}N_{l+1}$ to one l×6 matrix PC as in Equation (1).

(1) $$PC=\left\{\begin{array}{cccc} PM[N_{1}N_{2},P_{1}] & PM[N_{1}N_{2},P_{2}] & \cdots & PM[N_{1}N_{2},P_{6}]\\ PM[N_{2}N_{3},P_{1}] & PM[N_{2}N_{3},P_{2}] & \cdots & PM[N_{2}N_{3},P_{6}]\\ \vdots & \vdots & \ddots & \vdots \\ PM[N_{l}N_{l+1},P_{1}] & PM[N_{l}N_{l+1},P_{2}] & \cdots & PM[N_{l}N_{l+1},P_{6}] \end{array}\right\},$$

where $PM[N_{i}N_{i+1},P_{j}]$ is the physical structural properties listed in Table 1, $N_{i}N_{i+1}$ shows one 2-permutation of nucleotides located at position *i* and i+1 of sequence *L*, and $P_{j}$ means one physicochemical property.

### 2.2. k-Gram

The *k*-gram [38,39] is a pair of values (v,c), in which *v* represents one feature and *c* is the count of this feature. For analyzing the DNA sequence, the *v* can be defined as a combination of several units of nucleotides, and *c* is the number of combinations occurred in the sequence. For example, to represent a DNA sequence with 3-gram, *v* belongs to the set of combination of 3 nucleotides and *c* is the count of this combination in the whole sequence.

We utilize *k*-gram to extract the sequence features, and the nucleotide combination list *G* can be expressed as follows.

(2) $$\begin{array}{cc} G & =G_{1}\cup G_{2}\cup G_{3}\\ & =\left\{N_{i}\right\}\cup \left\{N_{i}N_{j}\right\}\cup \left\{N_{i}N_{j}N_{k}\right\}\\ & =\{A,C,G,T,AA,AC,\ldots ,TT,AAA,AAC,\ldots ,TTT\}, \end{array}$$

where $G_{1}$ is 1-gram with 4 features, $G_{2}$ is 2-gram with 16 features, $G_{3}$ is 3-gram with 64 features, $N_{i},N_{j},N_{k}\in \left\{A,C,G,T\right\}$.

Using *k*-gram to represent DNA segments (84 features), we can get the basic and intuitive information of the sequence with simple statistical methods. If two segments are more like each other and have more similar function, then their compositions may be more consistent. The number of each nucleotide and their combinations in a segment can directly reflect its composition.

### 2.3. Multivariate Mutual Information

Multivariate mutual information (MMI) has been used in many previous works [40,41,42] to extract features from sequence data. Therefore, the nucleotides sequence can also be represented using MMI. Inspired by the previous research works [29,43], we propose an improved method to extract features of the nucleotides sequence.

In order to use multivariate mutual information on a DNA sequence, we first define 2-tuple nucleotide composition set $T_{2}$ and a 3-tuple nucleotide composition set $T_{3}$ as follows.

(3) $$\begin{array}{cc} T_{2}= & \left\{AA,\;AC,\;AG,\;AT,\;CC,\;CG,\;CT,\;GG,\;GT,\;TT\right\}\\ T_{3}= & \{AAA,\;AAC,\;AAG,\;AAT,\;ACC,\;ACG,\;ACT,\;AGG,\;AGT,\;ATT,\\ & CCC,\;CCG,\;CCT,\;CGG,\;CGT,\;CTT,\;GGG,\;GGT,\;GTT,\;TTT\}. \end{array}$$

MMI in a tuple does not have a relationship with the order of nucleotides, so if two tuples have same constant but with different order, they may have he tsame information and be assigned as one type tuple. We can see from $T_{2}$ and $T_{3}$ that there does not exist the same composition with different order tuples, having 10 elements in $T_{2}$ and 20 elements in $T_{3}$.

We define 2-tuple mutual information for the elements in $T_{2}$ as follows:

(4) $$\begin{array}{cc} I(N_{1}N_{2})= & f\left(N_{1},N_{2}\right)ln\frac{f(N_{1},N_{2})}{f\left(N_{1}\right)f\left(N_{2}\right)}. \end{array}$$

We also define 3-tuple mutual information for the elements in $T_{3}$ as follows:

(5) $$\begin{array}{cc} I(N_{1}N_{2}N_{3}) & =f\left(N_{1},N_{2}\right)ln\frac{f(N_{1},N_{2})}{f\left(N_{1}\right)f\left(N_{2}\right)}+\frac{f(N_{1},N_{3})}{f(N_{3})}ln\frac{f(N_{1},N_{3})}{f(N_{3})}\\ & -\frac{f(N_{1},N_{2},N_{3})}{f(N_{2},N_{3})}ln\frac{f(N_{1},N_{2},N_{3})}{f(N_{2},N_{3})}. \end{array}$$

For a specific segment, $f(N_{i})$ is the frequency of nucleotide $N_{i}$ occurring in this segment, as $f(N_{i},N_{j})$ and $f(N_{i},N_{j},N_{l})$ are the frequency of 2-tuple and 3-tuple, respectively.

We use *M* to denote the multivariate mutual information feature set (30 features) as follows:

(6) M={I(AA),I(AC),⋯,I(TT),I(AAA),I(AAC),⋯,I(TTT)}.

### 2.4. Discrete Wavelet Transform

**Figure 2:**
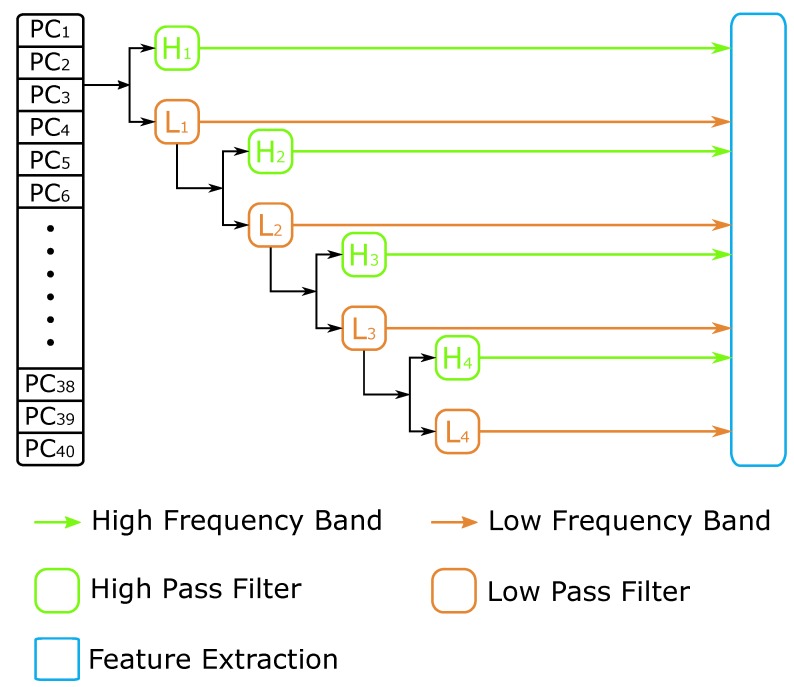
The discrete wavelet transform process.

Discrete Wavelet Transform (DWT) [34] is a transform operation where the wavelets are discretely sampled, and it can capture both the frequency and location information [44]. This transform is the projection of signal onto the wavelet function. When applied to DNA sequence analysis, DWT can decompose the physicochemical properties of the nucleotides sequence into a list of coefficients at different resolutions, and also removes the noise information from the high-pass profiles [30,45]. Figure 2 is an example of 4-level discrete wavelet transform. At every level, the data can be split into a high-frequency band containing more noisy information and a low-frequency band including more useful signal, and should be transformed at the next level.

At each level of DWT, the high-frequency band and low-frequency band signals are split. We calculate the maximum, minimum, mean, and standard deviation values of each band. Additionally, the first 5 elements are more significant information to represent the sequence in the compressed low-frequency band. Then, we can get 4+4+5 features from each level of DWT, and there are 52 features for 4-levels in the whole transformation process. Within the physical property matrix PC, we can extract 52 features for each property by using a 4-level DWT method. All 6 physicochemical properties can get 312 features. We use the symbol *D* to denote this DWT feature vector.

### 2.5. Pseudo Amino Acid Composition

Pseudo Amino Acid Composition (PseAAC) [35] is a very common method used to analyze protein sequences [31], and has been widely used in many research fields [36,46,47]. We adapt PseAAC by using physicochemical properties and correlation between each tuple to fit the DNA methylation prediction.

In the physical property matrix PC, there are 6 physicochemical properties and each one has 40 values that represent the physicochemical properties of the 2-tuple at the specific position. We use $PC_{i}$ to denote the *i*-th column vector, and we can define the PseAAC feature as follows.

(7) $$p_{i,\lambda }=\sum_{n=1}^{40-\lambda }\left(PC_{i}\left(n\right)-\bar{PC_{i}}\right)\left(PC_{i}\left(n+\lambda \right)-\bar{PC_{i}}\right),$$

where λ is the parameter of pseudo composition, *i* is the index of column in matrix PC, $PC_{i}\left(n\right)$ is the *n*-th value in the *i*-th column of matrix PC, and $\bar{PC_{i}}$ is the mean value of all elements in the *i*-th column.

We use λ from 0 to 30, which means that λ=0,1,…,30. Therefore, each column of PC can be produced as 31 features. All 6 physicochemical properties (186 features) can be calculated as follows:

(8) $$\begin{array}{c} P=\{p_{1,0},p_{1,1},\cdots ,p_{1,30},p_{2,0},\cdots ,p_{6,30}\}. \end{array}$$

### 2.6. Sparse Bayesian Classifier

Sparse Bayesian learning is a general Bayesian framework that can obtain sparse solutions to regression and classification tasks [48]. It uses Bayesian inference to obtain parsimonious solutions. With a probabilistic Bayesian learning framework, we can construct an accurate prediction model which offer a number of advantages over SVM, such as less computation complexity and probabilistic predictions.

In our method, we use the 612-D features to train a sparse Bayesian classifier. The 612-D features include 84-D from *k*-gram, 30-D from MMI, 312-D from DWT, and 186-D from PseAAC. After training, the classifier can be used to predict the methylation state of anDNA sequence. Especially, we adjust the parameters of the classifier to get better performance, as the training kernel of the sparse Bayesian learning model is a Gaussian kernel function, and the model’s iteration time is set to 5000.

## 3. Results

Our method can utilize the 612-D features to train a classifier and perform DNA methylation sites prediction. In order to see how well it works for methylation prediction, we tested it on a benchmark dataset and two scBS-seq datasets. On the benchmark dataset, we analyzed the importance of each feature, and then compared our method with iDNA-Methyl, Methylator, and MethCGI. For the scBS-seq datasets, DeepCpG [27] and another method proposed by Zhang et al. [26] were used in the comparison.

### 3.1. Datasets

#### 3.1.1. Benchmark Dataset

There are many databases collecting data about methylation or deregulation. These databases provide valuable resources for researchers to do computation experiments [49]. The database MethDB [32] provides data about methylation sequences. It focuses on the methylated cytosines in the DNA, and currently contains 20,236 records of 5-methyl-cytosine content data as well as 6312 records of individual patterns/profiles. MethDB has been used as a benchmark dataset by many methylation predictors, like iDNA-Methyl, Methylator, MethCGI, and so on. So, we use this dataset to test and analyze our method.

We extracted DNA sequences from the MethDB to construct positive and negative datasets. The extraction process is the same as the one used by iDNA-Methyl, in which the extracted sequence has 41 base pairs and the centering nucleotide is cytosine. Based on the methylation status of the sequences’ center cytosine, we divided the sequences into a methylated set (positive dataset) and a non-methylated set (negative dataset).

Among those extracted sequences, some of them were identical or very similar to each other. The redundancy affects the accuracy of prediction, so we used CD-HIT [50] to reduce the redundancy of datasets. According to the results of CD-HIT, if a sequence is identical to another one in the same dataset, one of these two sequences are removed. If two sequences from different dataset are identical (i.e., they are conflicting with each other), then both of them are removed. After reducing redundancy and removing conflict, there were 787 positive sequences and 1639 negative ones.

The size of the negative samples was two times that of the positive ones. This imbalanced situation of the datasets reduces the prediction’s accuracy. In order to prevent the false negative situation created by mis-predicting methylated sequences as non-methylation, we applied the SMOTE algorithm [51] to enlarge the positive dataset [52]. SMOTE can introduce synthetic samples by joining a real sample and its *k*-nearest neighbor. After the balancing process, 852 generated samples were added to the positive dataset. Because SVM is widely used and can achieve more accurate prediction than other machine learning models, we used SVM to do classification, as the final size of samples was not very large—both were 1639 in positive and negative.

#### 3.1.2. scBS-seq Dataset

In order to know the genome-wide prediction performance of our method, we applied it on 2 scBS-seq profiled cell datasets, which were 2i-cultured mESCs and serum-cultured mESCs. The 2i-cultured dataset is from 12 2*i*-cultured mESCs and the serum-cultured dataset is from 18 serum-cultured mESCs [33]. According to the CpG site’s position in the raw data file, we extracted sequences from the reference GRCm38 mouse genome. The extracted sequence has 201 base pairs with 100 before the site and 100 after it. Each CpG site’s methylation state is labeled according to the mapped reads. If one CpG site’s mapped reads in the raw data file had more tagged as methylated, then this site was labeled as methylated, and otherwise it was labeled as un-methylated. More specifically, the sites whose reads number were less than 4 should be discarded.

From the 2i-cultured mESCs, we got 123,680 methylated CpG sites, and 532,448 un-methylated CpG sites. The serum-cultured mESCs had 586,464 methylated sites and 833,779 un-methylated sites. Then, we could generate positive and negative datasets using the sequences around those sites, respectively. Unlike the benchmark dataset mentioned before, these two generated scBS-seq datasets are very large in sample size. It is not realistic to remove redundancy or balance the positive and negative samples. So, we applied our method on those original datasets without other processing.

### 3.2. Evaluation Method

#### 3.2.1. Target-Jackknife Cross-Validation

As a re-sampling technique, Jackknife is very useful for variance and bias estimation [53]. The basic procedure of Jackknife cross-validation is that it iteratively singles out one sample from the dataset, then uses the remaining samples to predict the singled-out sample. So, we can get a list of prediction results by using a Jackknife test and then calculating the evaluation variables. Jackknife cross-validation is suitable to test predictors on small datasets, so we chose jackknife cross-validation to evaluate our method’s performance on the benchmark dataset.

When generating the benchmark dataset, we used the SMOTE algorithm to increase the size of the positive set. Those additional hypothetical positive samples are not from the real world. In order to correctly evaluate our method, we filtered out those synthetic ones when doing the test, meaning that the ones created by SMOTE were only involved in the training but not the testing process. This is target-jackknife cross-validation. As there were 787 samples in the original positive dataset and 1639 in the negative one, we did 2426(787+1639) rounds of training and prediction, then calculated the AUC, MCC, etc.

#### 3.2.2. Holdout Validation

The sizes of scBS-seq datasets are very large. Using jackknife cross-validation is very time-consuming. So we chose holdout validation. Holdout validation is a type of simple validation. In this validation method, the samples are randomly assigned to two sets: the training set and the test set. The size of each set is arbitrary.

In our experiment, to simplify the procedure of dividing, we used samples from chromosomes 1, 3, 5, 7, 9, and 11 as the training set and samples from chromosomes 2, 4, 6, 8, 10, and 12 as the test set. Under this strategy, the 2i-cultured dataset had 64,793 positive samples and 270,550 negative samples used to train the model, and the number of samples used to test were 58,887 and 261,898, respectively. The constitution of the serum-cultured dataset was 306,836 positives and 432,339 negatives in the training set, and 279,628 positives and 401,440 negatives in the test set.

#### 3.2.3. Evaluation Criteria

To test the accuracy in predicting methylation sites, we used four statistical measurements to define its performance and effectiveness, as follows:

$$\begin{array}{cc} ACC & =\frac{TP+TN}{TP+FP+TN+FN},\\ MCC & =\frac{TP\times TN-FP\times FN}{\sqrt{\left(TP+FP)(TP+FN)(TN+FP)(TN+FN\right)}},\\ SN & =\frac{TP}{TP+FN},\\ SP & =\frac{TN}{TN+FP}. \end{array}$$

where true positive (TP) is the number of methylated segments that were correctly predicted as methylated sites, true negative (TN) is the number of non-methylated segments that were correctly predicted as non-methylated sites, false positive (FP) is the number of non-methylated segments that were incorrectly predicted as methylated sites, and false negative (FN) is the number of methylated segments that were incorrectly predicted as non-methylated sites. ACC means the overall prediction accuracy, MCC means Matthew’s correlation coefficient [54], SN and SP are sensitivity and specificity, respectively [55].

Besides those four criteria, we also used area under the receiver operating characteristic curve (AUC) [56] to analyze and evaluate our method. The receiver operating characteristic graph is created by plotting true positive rate against the false positive rate at various threshold settings [55]. A higher AUC value means that the classifier is scoring a positive instance greater than a negative instance—in other words, that this classifier is more efficient and accurate. So, the AUC can reflect the accuracy of prediction results.

### 3.3. Feature Analysis

The contribution of features to the classifier are not the same. In order to know the importance of each feature, we ran experiments using different combinations of features and evaluated the trained classifier. This experiment was performed on the benchmark dataset.

The experimental results are listed in Table 2. Firstly, we used the individual feature set to train the classifier separately, and used target-jackknife cross-validation to test it. The *k*-gram counts the occurrence of each nucleotide and their combination. It can get 84 features. Those features are the simplest way to represent a nucleotide sequence. Using this 84-D feature, the classifier achieved 0.73 on ACC, 0.32 on MCC, 0.35 on SN, 0.91 on SP, and 0.7143 on AUC. The MMI can get 30 features. They reflect the mutual information in the sequence. This representation form is a little more complex than *k*-gram, and contains deeper information. With this 30-D feature, the classifier was not as good as the *k*-gram one, but had an improvement of 0.0109 on SP. Among the four individual feature sets, the 312-D feature of DWT trained the best classifier with AUC reaching 0.8063, and the ACC, MCC, and SN were 0.7593, 0.4213, and 0.5057, respectively. DWT decomposes sequences into coefficients at different resolution, and also removes the noise information. So, it has better performance than other extraction methods. However, its SP value was the worst among the four individual feature sets, only reaching 0.8810 while the other three individual features had SP values greater than 0.9. From those results, we can see that DWT had a significant increase in AUC (at least 0.092), MCC (at least 0.10), and SN (at least 0.15) compared to the other individual features.

We also combined four features (612-D) together to train the classifier, and the result achieved 0.8338 on AUC, 0.77 on ACC, 0.45 on MCC, 0.55 on SN, and 0.87 on SP. The combination was better than four individual features when evaluated on AUC, ACC, MCC, and SN. However, for the SP value, the combination was not very good, as its result was 0.0482 less than the best one (0.9256 by PseAAC).

**Figure 3:**
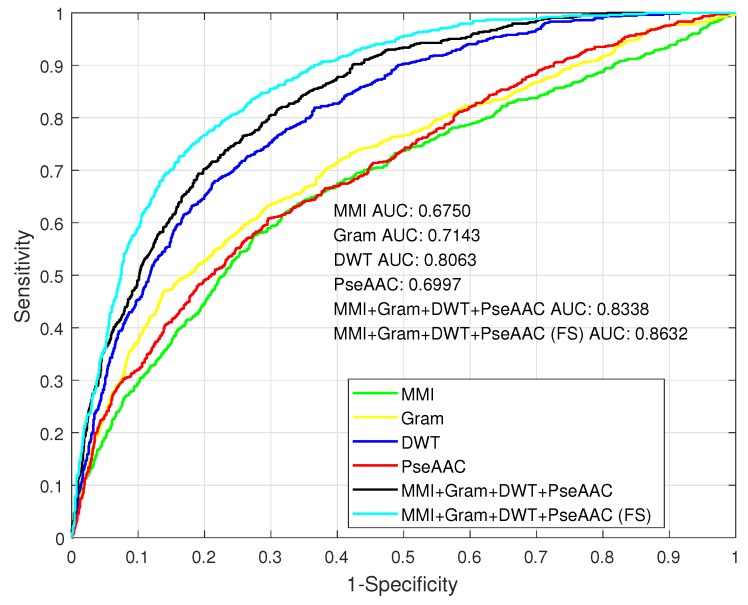
The receiver operating characteristic (ROC) of classifiers by using different features.

We plotted the receiver operating characteristic (ROC) curves of each classifier trained by different features or their combination in Figure 3. It is well-known that the area under the curve can be used to evaluate a classifier’s ability, and a larger area means better results. We can see that the DWT feature was the best one among four individual features. We found that MMI, *k*-gram, and PseAAC did not have very much difference—at least in terms of ROC. The curve of the combination was better than the DWT’s, but the improvement was not very significant. The AUC value of the combination feature was 0.8338.

### 3.4. Feature Selection

From the analysis of feature importance, we can see that some of the features were more important to the classifier than others. So, in the 612-D feature vector of the four features’ combination, some items were important and some were less effective. We wanted to use a feature selection algorithm [57] to choose the most useful features [58], and see the performance results of a classifier trained by those features. In this experiment, the benchmark dataset was used to train and test our method, and we used SVM to do classification.

**Figure 4:**
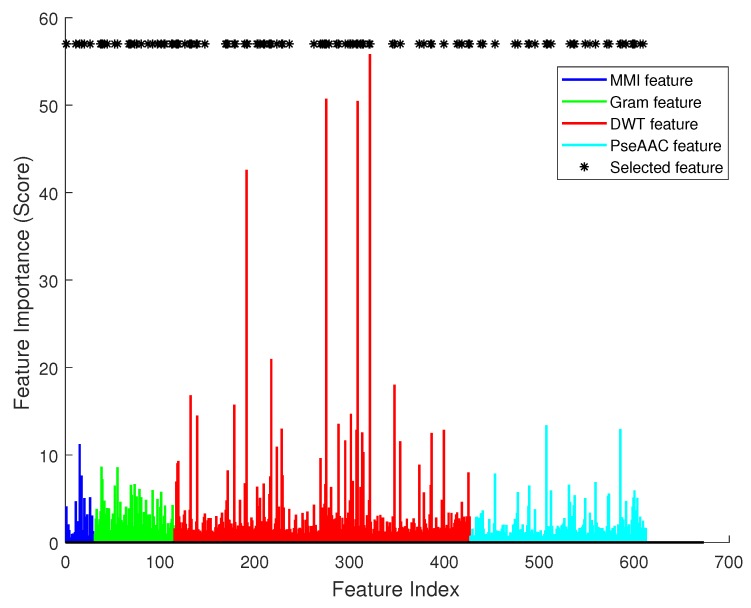
The importance score of each feature.

Figure 4 illustrates the effect of each single feature item. In this figure, there are 612 bars in total. The *x* axis is the index of each feature, and the *y* axis is the importance score calculated using SVM’s feature selection function. Asterisks mark the most significant features in feature selection. We can see that they had varied importance to the classifier, and obviously DWT had most significant effect (the red color bars).

**Figure 5:**
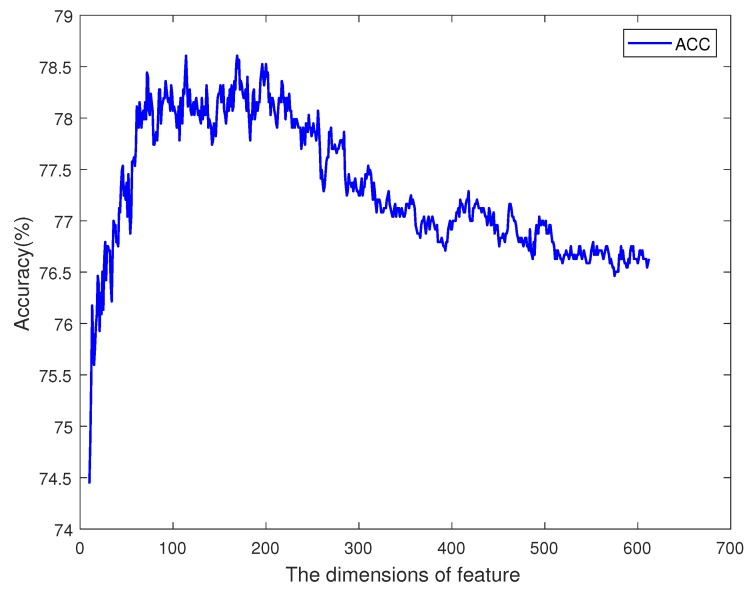
The tendency of accuracy on dimensions of features.

Then, we sorted the feature items by their importance scores, and iteratively increased the size of the feature vector. For every iteration, the most important one of the remaining feature items should be added to the vector, and a classifier can be trained. So, there were 612 classifiers, and each had a specific accuracy value. The tendency of accuracy is plotted in Figure 5. The *x* axis is the dimension of the feature, and the *y* axis is the accuracy of the classifier. When the dimension of the feature was less than 100, increasing the feature size could improve the accuracy, but when the dimension was greater than 200, the performance of the classifier with more feature items was worse. Between dimensions 100 and 200, there was an optimal plateau. Among 612 sorted features, the first 100 ones contained different information and attributes of the sequence, so increasing the size of the feature set could provide more support to the classifier. The features from the 100th to 200th contained information which may also be included in the first 100 features. This information does not have a great effect on the prediction result. So, the accuracy kept in a certain range from 77.5 to 78.5. The other remaining features may have had noise information, and affected the classifier’s performance. So, the curve in Figure 5 shows an increase at first, and remains high for a period after reaching the highest point, then it decreases. The most accurate classifier was trained by using 114 significant items, which are marked in Figure 4 by an asterisk symbol. In the optimal feature vector, 5 items were selected from feature MMI, 21 were from k-gram, 64 were from DWT, and 24 were from PseAAC.

We used the the 114-D selected feature to train a classifier, and the performance result is listed in Table 2. From the table we can see that this classifier could achieve 0.8632 on AUC, 0.8017 on ACC, 0.5558 on MCC, 0.7268 on SN, and 0.8377 on SP. Especially, with feature selection, the AUC value could increase by 0.03 than without feature selection. We plotted the ROC of this classifier in Figure 3. The curve of the selected feature is prominent, and has significant changes from the curve of the combination feature. So, feature selection is useful in our method.

### 3.5. DWT Feature Analysis

According to Figure 4, we can see that DWT features are more important than other ones in our method. We used only DWT features to train an SVM classifier and applied feature selection on them. Then, we analyzed those features further to see the properties of DWT feature extraction. This experiment was done on the original unbalanced benchmark dataset.

**Figure 6:**
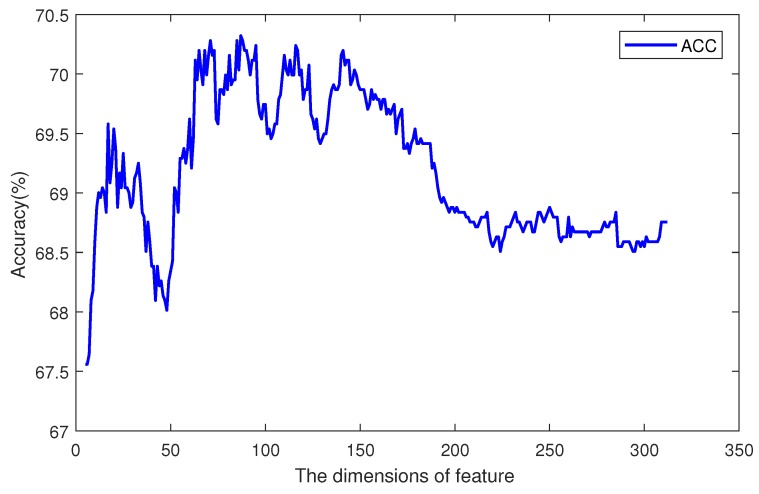
The tendency of accuracy on DWT features.

The accuracies of different dimensions are plotted in Figure 6. The *x* axis is the dimension of DWT features, and the *y* axis is the accuracy of prediction. The total dimension of features was 312. When the dimension was 87, the accuracy reached its max value. Before point 87, the main tendency was increasing, but with a small fluctuation around point 50. After point 150, the accuracy decreased along with more features.

We analyzed these selected 87 features and found that different levels of DWT had different importance. There were 4 levels of extraction, and each level could get 78 features. With feature selection, each level contributes a different number of features. Among the 78 features from the 1st-level, 31 were selected. Selected features from the 2nd- to 4th-levels were 15, 20, and 21, respectively. From the constitution, we can see that the first level had the greatest importance and the second level was the least important level. The effects of third and fourth levels had no great difference. Those properties can help us to optimize the DWT in future works.

### 3.6. Performance on Benchmark Dataset

With the utilization of feature selection in our method, we compared it with three existing methods: MethCGI [24], Methylator [19], and iDNA-Methyl [21]. This comparison is based on the benchmark dataset, and our method uses SVM as classifier. The results are listed in Table 3.

Among the three reference methods, iDNA-Methyl was the best, and ACC, MCC, SN, and SP were 0.77, 0.54, 0.61, and 0.90, respectively. iDNA-Methyl had great improvements over the other two methods: ACC increased by 0.0366 and MCC by 0.1723. Our method had a better result than those three methods in ACC, MCC, and SN. The ACC of our method was 0.8017, with 0.0268 increment to iDNA-Methyl. The MCC of our method was 0.55, which is also better than the other methods, but the improvement was not very obvious. The SN of our method had a very significant value (0.7268) compared to iDNA-Methyl (0.61) and Methylator (0.51). When compared to other methods on SP, we could only achieve 0.8377. This value was slightly lower than iDNA-Methyl’s 0.9033 and MethCGI’s 0.8542. The Methylator’s SP was only 0.8078, so our method remained better than Methylator. From these results, we can see that our method solved some of the limitations of existing methods and could get more accurate results.

### 3.7. Performance on scBS-seq Dataset

We also tested our method on scBS-seq profiled cells’ data. In our experiment, two datasets were used: 2i-cultured and serum-cultured. For each dataset, we trained a list of classifiers using each single cell’s training set, and tested the classifier with the corresponding cell’s test set. The best accuracy of all the classifiers could be used as the result of our method on that dataset. Considering the large size of the samples, we used sparse Bayesian learning rather than SVM to do classification, and used hold validation to evaluate our method.

**Figure 7:**
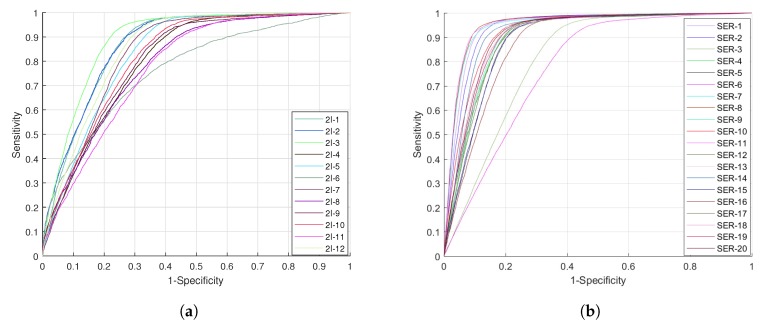
The ROC of our method on different cells. (**a**) The ROC on 2*i*-cultured mESCs. (**b**) The ROC on serum-cultured mESCs.

Figure 7 shows the the ROCs of our method on two datasets. Each ROC in the figure is a performance result of our method when it was trained and tested on a specific cell’s data. From the curves, we can see that in the 2i-cultured dataset, the curves were focused in a small region, and there was not a very large difference between each cell’s result. In the serum-cultured dataset, except for the two curves of SER-3 and SER-6, the other curves were very consistent. Since the methylation pattern of SER-3 and SER-6 deviated strongly from the remaining serum cells [27,33,59], the curves of those two cells deviated from the normal ones.

The results of our method and other methods are listed in Table 4. DeepCpG, which uses a deep neural network, was proposed by Angermueller et al. [27]. Another method, proposed by Zhang et al., uses genome annotation marks as feature and random forests as classifier [26], and we refer to it as RF_Zhang. All of the values in Table 4 are the best results of each method on the specific dataset.

From Table 4, we can see that our method was comparable with the other two methods. On dataset 2i-cultured cells, the AUC of our method was 0.8896 while DeepCpG and RF_Zhang could only reach 0.8497 and 0.8134, respectively. Besides the 0.04 improvement on AUC, our method was also better on ACC (0.9168) and SP (0.9987). On the dataset of serum-cultured cells, our method’s performance was outstanding. The AUC of our method reached 0.9511, an increase of 0.0274 from DeepCpG’s. Additionally, the MCC and SN had improvements of 0.0521 and 0.1541. The ACC value was almost the same as DeepCpG’s.

## 4. Discussion

### 4.1. Contribution of Method

From the experimental results, we know that our method—especially the four feature extraction methods proposed in this research work—is effective in extracting information from sequence context and physical structure. Our method can use the benefit of the DNA sequence’s original constitution, unlike other methods that use functional expression to construct features. Additionally, the high accuracy of our method’s prediction results shows that the sequence around the candidate site is important to predict its methylation states. From the feature selection experiment, we found that DWT and PseAAC had more contribution to the classifier than the other two feature extraction methods. They generated features from the physicochemical property. So, whether or nota site can be methylated is affected very significantly by the physical structure around it.

### 4.2. Method Feasibility

On huge datasets, the running time used by a method is an important factor to evaluate its practicality. To get an intuitive understanding of the computational complexity of our method, we implemented it using MATLAB^®^ script and executed it on a Think Station P700 computer. This computer had two 12 core Intel^®^ Xeon^®^ E5 CPUs and 320 G RAM. The clock rate of the CPU was 2.40 GHz. Specifically, our program used 1 core and less than 10 G memory.

We roughly measured the running time used by each feature extraction method, listed in Table 5. From those results, we can see that *k*-gram algorithm was the simplest one and could process 10 thousand sequences in 19.25 s. The MMI and DWT methods were more complex, as their execution time was more than 280 s to process the same sequences. This complexity was 15 times that of *k*-gram’s. Compared to MMI and DWT, PseAAC was fast, even if its running time was 82.13 s per 10 thousand.

When training the classifier, the sparse Bayesian learning algorithm could learn 3500 sequences per second. This is very fast. This means that for a dataset size of one million, our method could finish the training process in 5 min. This low complexity makes our method suitable for use in practical applications.

## 5. Conclusions

In this paper, we propose a novel method for DNA methylation prediction. We designed and implemented four feature extraction methods to extract sequence and physical structure information, then used sparse Bayesian learning to do classification. The four feature extraction methods were *k*-gram, multivariate mutual information, discrete wavelet transform, and pseudo amino acid composition. Besides those, we also analyzed the importance of each feature, and used feature selection to improve the performance of our method. Testing on three datasets (a benchmark dataset and two scBS-seq datasets) demonstrated that our method is outstanding. The AUC values on the three datasets were 0.8632, 0.8896, and 0.9511. When compared with other methods, our method’s AUCs on the three datasets were the greatest, and we achieved great improvements of 0.02 to 0.04. Especially on the benchmark dataset, our method had 0.8632 on AUC, 0.8017 on ACC, 0.5558 on MCC, and 0.7268 on SN. Those four values are the greatest among all the methods. According to the evaluation value and comparison, our method was effective in predicting DNA methylation sites. Furthermore, the feature extraction methods used in our method are helpful for other prediction problems.

## Figures and Tables

**Table 1:** The original values of six physical structural properties.

2-Nucleotides	Twist	Tilt	Roll	Shift	Slide	Rise
AA	0.026	0.038	0.020	1.69	2.26	7.65
AC	0.036	0.038	0.023	1.32	3.03	8.93
AG	0.031	0.037	0.019	1.46	2.03	7.08
AT	0.033	0.036	0.022	1.03	3.83	9.07
CA	0.016	0.025	0.017	1.07	1.78	6.38
CC	0.026	0.042	0.019	1.43	1.65	8.04
CG	0.014	0.026	0.016	1.08	2.00	6.23
CT	0.031	0.037	0.019	1.46	2.03	7.08
GA	0.025	0.038	0.020	1.32	1.93	8.56
GC	0.025	0.036	0.026	1.20	2.61	9.53
GG	0.026	0.042	0.019	1.43	1.65	8.04
GT	0.036	0.038	0.023	1.32	3.03	8.93
TA	0.017	0.018	0.016	0.72	1.20	6.23
TC	0.025	0.038	0.020	1.32	1.93	8.56
TG	0.016	0.025	0.017	1.07	1.78	6.38
TT	0.026	0.038	0.020	1.69	2.26	7.65

**Table 2:** The performance ${}^{1}$ of our method by using different features.

FEATURE	AUC	ACC	MCC	SN	SP
*k*-Gram	0.7143	0.7312	0.3288	0.3532	0.9128
MMI	0.6750	0.7061	0.2430	0.2529	0.9237
DWT	0.8063	0.7593	0.4213	0.5057	0.8810
PseAAC	0.6997	0.7214	0.2936	0.2961	**0.9256**
Combination ${}^{2}$	0.8338	0.7725	0.4589	0.5540	0.8774
Combination (FS) ${}^{3}$	**0.8632**	**0.8017**	**0.5558**	**0.7268**	0.8377

${}^{1}$ The values were calculated using the testing results on benchmark dataset. The classifier was support vector machine (SVM), and the validation method was target-jackknife cross-validation. ${}^{2}$ Feature size was 612-D, including all of *k*-gram, MMI, DWT, and PseAAC. ${}^{3}$ Feature size was 114-D feature, selected by feature selection in SVM. AUC: area under the receiver operating characteristic curve; ACC: accuracy; MCC: Matthews correlation coefficient; SN: sensitivity; SP: specificity; FS: feature selection. The bold digits are the greatest values in each column.

**Table 3:** Comparison of our method, MethCGI, Methylator, and iDNA-Methyl on the benchmark dataset.

Predictor	ACC	MCC	SN	SP
iDNA-Methyl	0.7749	0.5471	0.6125	**0.9033**
Methylator	0.7135	0.3327	0.5172	0.8078
MethCGI	0.7383	0.3748	0.4968	0.8542
Our Method ${}^{1}$	**0.8017**	**0.5558**	**0.7268**	0.8377

${}^{1}$ Feature size is 114-D feature, selected by feature selection in SVM. Our method used SVM classifier and target-jackknife cross-validation. The bold digits are the greatest values in each column.

**Table 4:** Comparison of our method, DeepCpG, and RF_Zhang on scBS-seq profiled mESCs.

	2*i*-Cultured Cells ${}^{1}$					Serum-Cultured Cells ${}^{2}$				
	AUC	ACC	MCC	SN	SP	AUC	ACC	MCC	SN	SP
DeepCpG	0.8497	0.7752	**0.5514**	**0.7545**	0.8351	0.9237	0.9198	0.7681	0.7907	0.9608
RF_Zhang	0.8134	0.7610	0.5452	0.6809	0.9234	0.9084	**0.9225**	0.7634	0.7704	**0.9680**
Our Method ${}^{3}$	**0.8896**	**0.9168**	0.5221	0.6323	**0.9987**	**0.9511**	0.9106	**0.8202**	**0.9448**	0.9034

${}^{1}$ For all the three methods, the results are using the best value of the 12 cells. ${}^{2}$ For all the three methods, the results are using the best value of the 18 cells. ${}^{3}$ Our method used sparse Bayesian learning classifier and holdout validation. The bold digits are the greatest values in each column.

**Table 5:** Running time of each feature extraction method.

	Feature Sets			
	*k*-Gram	MMI	DWT	PseAAC
Running Time (s/10 K sequences)	19.25	280.17	287.31	82.13

## Abbreviations

The following abbreviations are used in this manuscript:

AUC	area under the receiver operating characteristic curve
ACC	accuracy
DNA	deoxyribonucleic acid
DWT	discrete wavelet transform
MCC	matthew’s correlation coefficient
MMI	multivariate mutual information
SN	sensitivity
SP	specificity
SVM	support vector machine
PseAAC	pseudo amino acid composition
mESCs	mouse embryonic stem cells
scBS-seq	single-cell bisulfite sequencing
